# Nourishing Physical Productivity and Performance On a Warming Planet - Challenges and Nutritional Strategies to Mitigate Exertional Heat Stress

**DOI:** 10.1007/s13668-024-00554-8

**Published:** 2024-07-12

**Authors:** Alan J. McCubbin, Christopher G. Irwin, Ricardo J. S. Costa

**Affiliations:** 1 Department of Nutrition, Dietetics and Food, Monash University, Level 1, 264 Ferntree Gully Road, Notting Hill, Victoria, 3168 Australia; 2 School of Health Sciences and Social Work, Menzies Health Institute Queensland, Griffith University, Gold Coast, QLD Australia

**Keywords:** Heat stress, Exertional heat illness, Body temperature, Hydration

## Abstract

**Purpose of Review:**

Climate change is predicted to increase the frequency and severity of exposure to hot environments. This can impair health, physical performance, and productivity for active individuals in occupational and athletic settings. This review summarizes current knowledge and recent advancements in nutritional strategies to minimize the impact of exertional-heat stress (EHS).

**Recent Findings:**

Hydration strategies limiting body mass loss to < 3% during EHS are performance-beneficial in weight-supported activities, although evidence regarding smaller fluid deficits (< 2% body mass loss) and weight-dependent activities is less clear due to a lack of well-designed studies with adequate blinding. Sodium replacement requirements during EHS depends on both sweat losses and the extent of fluid replacement, with quantified sodium replacement only necessary once fluid replacement > 60–80% of losses. Ice ingestion lowers core temperature and may improve thermal comfort and performance outcomes when consumed before, but less so during activity. Prevention and management of gastrointestinal disturbances during EHS should focus on high carbohydrate but low FODMAP availability before and during exercise, frequent provision of carbohydrate and/or protein during exercise, adequate hydration, and body temperature regulation. Evidence for these approaches is lacking in occupational settings. Acute kidney injury is a potential concern resulting from inadequate fluid replacement during and post-EHS, and emerging evidence suggests that repeated exposures may increase the risk of developing chronic kidney disease.

**Summary:**

Nutritional strategies can help regulate hydration, body temperature, and gastrointestinal status during EHS. Doing so minimizes the impact of EHS on health and safety and optimizes productivity and performance outcomes on a warming planet.

## Introduction

Climate change is anticipated to increase the frequency of exposure to ambient conditions that challenge human thermoregulatory capacity (i.e., heat stress) [1]. The impact of heat stress is especially significant for physically active populations (e.g., recreational and elite athletes, and individuals working in military, emergency services, building and construction, mining, forestry, farming and agricultural sectors, amongst others), since physical activity increases metabolic heat production, (i.e., exertional-heat stress (EHS)) [1, 2]. The predicted increase in extreme heat days [3] presents a substantial economic and health issue. Ambient temperatures (T_amb_) of 33–34 ºC are associated with a 50% reduction in labor productivity [4], reduced athletic performance, and increased risk of exertional heat illness (EHI) [5], a continuum of health outcomes from mild to potentially fatal, and usually but not always related to the extent and duration of core temperature elevation [5, 6]. Development of EHS relates not only to metabolic heat production, but protective clothing and equipment that reduces heat loss through convection and sweat evaporation, and for outdoor EHS, direct sun exposure that reduces non-evaporative heat loss [7]. EHS impacts an individual’s ability to maintain a stable, safe body temperature (usually < 39 ºC), and increases the risk of workplace injuries as a consequence of increased fatigue, impaired cognition and decision-making, EHI, and other negative health outcomes [8]. For those exercising in hot environments, EHS not only increases the risk of EHI, but can result in significantly increased sympathetic nervous system activity and reductions in splanchnic blood flow, increasing the risk of gastrointestinal [9] and renal [10] disturbances.

Nutritional factors can help mitigate EHS and prevent/minimize its consequences [11, 12]. Since several recent international sporting events have taken place in hot ambient conditions (e.g., 2016 UCI Road Cycling World Championships, 2019 World Athletics Championships, 2022 FIFA World Cup, 2020 Tokyo Olympic Games), there has been an increased research focus in physiology and nutrition to enhance safety and optimize performance during EHS. This narrative review summarizes key concerns, and provides an update on recent advances regarding nutrition-related prevention and management strategies for EHS in occupational and exercising populations.

## Specific Challenges in a Hot Environment

### Exertional-Heat Illness

The term EHI represents both mild and severe consequences of EHS [6]. The milder form, ‘heat exhaustion’, relates to loss of blood volume and pressure due to dehydration and/or blood flow redistribution to the skin, with subsequent symptoms of headache, nausea, vomiting, and/or possible syncope during or immediately following exercise [13]. ‘Heat injury’ describes a more severe outcome whereby hyperthermia has resulted in injury to tissue or organ systems, most commonly in the gastrointestinal tract, liver, kidneys, and skeletal muscles [6]. The most severe outcome is ‘exertional-heat stroke’, a potentially life-threatening condition characterized by severe central nervous system (CNS) dysfunction (i.e., altered behavior, seizure, loss of consciousness), translocation of pathogenic agents (e.g., bacteria and/or bacterial endotoxins) from the intestinal lumen into systemic circulation, and/or release into circulation of proteins and metabolites from skeletal muscle tissue, with subsequent systemic inflammatory responses and potential multi-organ failure [6]. Such outcomes have been reported in both athletic and occupational settings, and highlighted particularly in the US military (0.37 events per 1000 person-years) [14] and the construction industry (36% of all occupational heat-related deaths) [15]. The incidence of EHI reported during organized sport varies substantially, but ranges from virtually zero to as high as 55% of studied athletes completing a desert ultramarathon [16], and exertional heat stress specifically is the second most common cause of fatality in athletes aside from trauma [14]. Detailed epidemiology and pathophysiology of EHI is beyond the scope of this review, but readers are directed to recent, comprehensive reviews on the topic [6, 14].

### Exercise-Induced Gastrointestinal Syndrome

*‘Exercise-Induced Gastrointestinal Syndrome*- (EIGS)’ is a condition defined as symptomatic or asymptomatic disturbances to gastrointestinal epithelial integrity and/or function, with systemic consequences, in response to exercise stress. Such disturbances have been linked to outcomes ranging from minor symptomatic inconvenience, to systemic outcomes that can lead to fatality (i.e., sepsis and systemic shock). The etiology and pathophysiology of EIGS has been thoroughly explored previously [17–19]. In short, splanchnic hypoperfusion and gastrointestinal ischemia can result from redistribution of blood flow to skeletal muscles and peripheral circulation during EHS [20, 21]. Such circulatory adjustments may promote intestinal epithelial cell injury and hyperpermeability, and result in translocation of pathogens (e.g., whole bacteria and/or bacterial endotoxins) from the gastrointestinal lumen into circulation, prompting local and/or systemic inflammatory responses [22–26]. Impaired gastrointestinal motility, transit, digestive function, and nutrient absorption may also occur [17, 27–34], potentially via increases in stress hormone responses and sympathetic activation [28, 29, 34, 35]. Exercise in hot ambient conditions (e.g., ≥ 35.0 ºC), irrespective of relative humidity, has recently been confirmed to exacerbate EIGS and associated performance-debilitating gastrointestinal symptoms (GIS), more than other potential factors (e.g., exercise duration, intensity, modality, hydration status, biological sex, age, fitness status, anxiety, and/or gut microbiome) [9, 19]. In recent years, it has been confirmed that prolonged steady-state exercise (e.g., 2 h at 60% *V̇*O_2max_) in hot ambient conditions elicits a greater gastrointestinal integrity and systemic disturbance, compared with higher intensity interval exercise (2 h HIIT protocol at 55–80% *V̇*O_2max_) in temperate conditions [9, 22, 26, 36]. Further analysis confirms a core temperature ≥ 39.5 ºC represents the greatest risk for observing EIGS biomarkers and GIS of clinical relevance and concern [9, 26]. However, gastrointestinal integrity disturbances in response to EHS do not appear to directly translate to functional disturbances. Exploratory research suggests the T_amb_ exercise is performed in, and the individual core temperature response, will inform the risk of clinical outcomes related to EIGS; which may include, but are not limited to, acute ischemic colitis, sepsis, and systemic shock, and align with the pathophysiology of heat stroke [9, 37]. Additional causal pathways for EIGS have previously been proposed, although not specific to EHS. These included the mechanical strain of exercise on the splanchnic arena, with subsequent hypersensitivity of epithelial, connective, and/or surrounding tissues of the gastrointestinal tract [19, 38, 39]; and/or exaggerated metabolic responses, namely metabolic acidosis associated with high intensity exercise [40, 41], and hypoglycemia associated with prolonged strenuous exercise [42].

### Acute Kidney Injury

In addition to the gastrointestinal tract, both heat stress and exertional stress have separate and additive effects that increase the risk of acute kidney injury (AKI). Like the etiology of EIGS, this is thought to be due to a reduction in visceral blood flow, due to both sympathetic activation and increased core temperature [43]. The effect of EHS on the kidney is further exacerbated when blood volume is reduced due to inadequately replaced sweat losses [44], and/or in situations of significant muscle damage and associated release of myocellular contents into circulation (i.e., rhabdomyolysis) [45], mostly reported in unaccustomed physical exertion or ultra-endurance exercise [46]. Perhaps more concerningly, frequent exposure to AKI, mediated by EHS, is now suspected to be a major causal factor in the rise in chronic kidney disease (CKD) in occupational settings in Central America, where repeated and prolonged exposure to EHS without adequate mitigation strategies is common amongst agricultural workers [47]. While the mechanistic evidence describing the link from frequent AKI to CKD are not yet fully elucidated in humans, recent evidence suggests that frequent occurrences of renal ischemia, similar to that described with EIGS, has a major contributing role [48].

## Nutritional Strategies for Mitigating the Effect of Heat On Physical Performance and Productivity

### Fluid Balance

The most well-identified effect of EHS and its role in attenuating physical performance is the impact of thermoregulatory sweating, and subsequent body water and sodium deficits. Preventing large disturbances in body water balance is crucial in minimizing risk of EHI and optimizing physical performance and productivity in hot ambient conditions [5, 14, 49, 50]. Inability to prevent large body water deficits results in decreased blood volume and increased plasma osmolality [12], hindering the body's ability to regulate temperature, resulting in fatigue, and increased risk of EHI, AKI and EIGS [12, 27, 51]. Ongoing debate persists regarding the optimal fluid replacement strategy during EHS. Discussion often ties back to establishing a critical threshold at which dehydration impacts performance; with general consensus (predominantly from laboratory-based studies) that performance decline during EHS typically begins when fluid deficits ≥ 2% BM loss [50]. However, conventional research methods employed in this area enable participants awareness of the hydration conditions during activity (i.e., treatments often follow a ‘complete’ vs ‘no replacement’ paradigm, and lack participant blinding), which itself may influence outcomes [52]. Several recent investigations have attempted to blind participants regarding hydration intervention, employing intravenous saline infusion [53–55] or gastric administration of water [56–58]. These studies found that dehydration (> 2% BM loss) does negatively impact performance in hot conditions, regardless of blinding. However, a recent study using gastric delivery of water suggests that at lower levels of dehydration (i.e., < 2% BM loss), an individual’s perception of hydration intervention might hinder performance and perception of thirst [59]. It is important to take into consideration other contextual aspects such as the intensity and duration of physical tasks being undertaken, ambient conditions, availability/accessibility of fluids (and food) and capacity to consume them, and individual preferences, when formulating a hydration approach that is practical and achievable [60, 61].

A fundamental component of any hydration strategy involves monitoring hydration status and drinking adequate amounts of fluid in advance of EHS. Guidelines for pre-exercise hydration universally recommend individuals commence physical activity in a euhydrated state [62, 63]. Determining euhydration quantitatively across individuals can be challenging, as no single measure of body fluid compartment volume (e.g., total body water, extracellular fluid, or plasma volume), or solute concentration (e.g., serum or plasma osmolality) is considered definitive in all circumstances [64]. Similarly, renal responses to changes in these markers (e.g., urine specific gravity or osmolality) can provide a valid estimate of hydration status at rest (especially from first-morning urine samples), but are not considered valid in close proximity to acute changes in fluid balance (e.g., EHS and/or fluid intake) [64]. Instead, a more practical, multifactorial method of avoiding downward fluctuations in body mass, increased urine color and/or thirst perception in a rested state, was proposed almost twenty years ago [65], but has only very recently been validated for this purpose in free-living conditions, at multiple times of day, against both blood and urine biomarkers [66].

While the volume and type of fluids integrated into pre-activity hydration strategies should be determined by the individual's current hydration status (and other contextual factors), consuming 5–10 ml/kg BM 2–4 h prior to physical activity is usually sufficient [62]. Where practical constraints exist to consuming adequate fluid during EHS, individuals may contemplate pre-exercise hyperhydration. This has been a focus of research in the past two decades, and is achieved with a larger pre-exercise volume (~ 10–25 mL/kg BM) of water, combined with an osmotic agent (e.g., sodium chloride (NaCl), glycerol) to improve fluid retention and reduce diuresis [67]. Research focus on hyperhydration with osmotic agents has fluctuated over time, due to the addition in 2010, and subsequent removal in 2018, of glycerol from the World Anti-Doping Agency’s prohibited substances list. Only recently have such strategies been reviewed in their totality, with two recent systematic reviews noting that the effects of hyperhydration (regardless of the approach taken) are not uniform, that there are risks of experiencing gastrointestinal side effects from osmotic agents, and when physical performance benefits have been observed, they are typically modest [67, 68]. When practical constraints are not a limiting factor to optimal hydration practices during an activity, it appears unlikely that prior hyperhydration will mitigate EHI risk or improve performance.

Factors likely to influence fluid consumption during EHS include access to fluids, opportunities to drink, beverage temperature, and gastrointestinal tolerance to fluid volumes that limit total body water (TBW) deficits to < 2% BM [60]. In most circumstances, individuals can rely on physiological cues (i.e., thirst) to guide fluid intake, or consume fluid ad libitum (i.e., whenever and in whatever volume desired, without specific focus on thirst) [69], with most individuals able to comfortably drink 400–800 ml/h [50, 62]. However, in cases of extreme heat (i.e., T_amb_ > 38 ºC), thirst or an ad libitum approach to drinking may be inadequate to promote optimal fluid intake [69, 70]. As such, individualized and tailored fluid replacement plans based on prior evaluations of fluid balance, perceived thirst, gastrointestinal tolerance, and performance data (where appropriate) under comparable scenarios is ideal. Cool (< 22 ºC) or cold (< 10 ºC) beverages are often more appealing in hot conditions, and can promote higher voluntary fluid intakes [71], and improved thermal comfort [72, 73]. Keeping fluids cold can be a practical challenge in many situations, but prior planning (e.g., freezing drinks overnight for next day consumption) can be helpful in such circumstances. It should also be noted that excessive fluid intake that causes an increase from pre-exercise TBW is neither ergogenic nor recommended for athletes, due to the significantly increased risk of exercise-associated hyponatremia, and is a potential consequence in both athletic and occupational settings of strategies that encourage liberal fluid intake without regard for thirst perception or urine volume [74].

Guidelines for replenishing fluid losses post-EHS recommend individuals consume ∼125–150% of any remaining fluid deficit in the ~ 4 h following physical activity, to allow for some residual sweat and urinary losses [11, 62]. While any type of beverage contributes to fluid intake and recovery of fluid balance [75, 76], recommendations suggest avoiding certain alcoholic beverages due to their well-documented diuretic effect that results in a less positive net fluid balance post-exercise once alcohol content exceeds around 4% *w/v* [77, 78]. Some fluids (e.g., milk-based beverages) have superior fluid retention properties (i.e., a higher beverage hydration index), due to the presence of other nutrients (i.e., carbohydrate, protein, and/or sodium) [75]. Consumption of these nutrients, within a fluid or in food co-ingested with fluids, enhances fluid retention [79, 80], and may be particularly helpful for individuals struggling to consume large fluid volumes immediately after EHS, although a risk exists of greater post-exercise GIS, especially if they have experienced substantial thermoregulatory strain and/or impairment of gastrointestinal integrity/function [33].

### Sodium Balance

Limited guidelines exist to guide sodium replacement during EHS, and range from broad suggestions to replace ‘large sodium losses’ [62], to advocating sodium replacement only through foods and fluids according to cravings [63]. As the main electrolyte lost in sweat, sodium has received the most research attention, and recent publications have provided more context to recommendations. Its role in regulating TBW and movement of water between intracellular fluid (ICF) and extracellular fluid (ECF) relates to the dominant role of sodium in influencing ECF and plasma osmolality (P_Osm_), which in turn has the greatest influence on thirst and diuresis [81, 82]. Since sweat is hypotonic to plasma [83], unreplaced sweat losses during EHS increase P_Osm_ while reducing plasma volume (P_v_) and TBW. Drinking hypotonic solutions will lower plasma sodium concentration ([Na^+^]_plasma_) and therefore P_Osm_, but only when fluid replacement exceeds around 60–80% of losses [84]. The need for sodium replacement to influence [Na^+^]_plasma_ and P_Osm_ therefore is not only dependent on fluid and sodium losses, as has been traditionally emphasized, but also the extent to which fluid is replaced [85]. This insight suggests that prolonged sweat losses, while simultaneously replacing > 60–80% of fluid losses, is the only scenario during EHS in which purposeful, quantified sodium replacement is necessary to benefit hydration status, and therefore health, physical performance and/or productivity [85]. Consistent with this, studies of sodium replacement over 3–5 h EHS, either with a fixed fluid volume and arbitrary sodium replacement [86, 87], or personalized sodium replacement and ad libitum fluid intake [88], failed to show benefits of sodium on thermo-physiological strain. When both water and sodium replacement have been systematically manipulated during exercise, it is the change in TBW, not the balance between ICF and ECF, that influences core temperature and cardiovascular responses [89]. However, sodium intake may still play a role by influencing drinking behavior through beverage palatability and voluntary fluid intake, providing a useful strategy in situations where the athlete or worker is failing to meet their fluid replacement needs [61].

### Internal Cooling Strategies

The use of beverage temperature as a means of influencing core temperature, thermal perception, or both, has received increased research attention in the past decade. While most research on these interventions has a sports focus, internal cooling (i.e., cold fluids and ice slurries) in occupational settings has particular merit for its potential to reduce body temperature during rest periods, without the need to remove the protective clothing or equipment necessary for external cooling strategies (e.g., ice vests or cold water immersion) [90]. Consuming ~ 4.5–7.5 g/kg BM ice slurry at rest (i.e., pre-cooling),which acts as a heat sink due to the energy cost of transforming ice to water and raising it to body temperature, temporarily lowers core temperature by 0.1–0.8 ºC within ~ 30 min [91]. The number of studies investigating ice slurry pre-cooling has grown since 2010, with the totality of evidence now suggesting a beneficial physical performance effect for constant workload but not self-paced exercise performance, which are smaller effects than those achieved through external cooling methods like ice vests, cold water immersion, and limb cooling [91]. Ice slurry ingestion during EHS (per-cooling) has minimal effect on physical performance [91], likely because it causes compensatory responses in thermoregulatory sweating to maintain the same heat balance [92]. However, there may be benefits of internal per-cooling when evaporative heat loss is restricted, such as individuals with spinal cord or significant burn injuries [92], or when protective clothing prevents sweat evaporation, especially in lower intensity (~ 40% *V̇*O_2max_) activities where the lower rate of metabolic heat production can be more likely offset by the heat sink effect of ice ingestion, and may be more representative of some occupational and military settings [93]. Ingestion of ice during exercise or occupational activities is not always practical, however. For example, a field study of ice slurry ingestion in firefighters in a tropical climate found that they were unable to ingest the prescribed 7.5 g/kg, instead waiting for much of it to melt and then drinking the cool fluid, with only small effects on core temperature [94]. Cold water rather than ice can still have a small effect on core temperature and cortisol response when consumed during moderate intensity (~ 60% *V̇*O_2max_) exercise compared to ambient temperature water [95], and thus may represent a more practical alternative in settings where ice ingestion is impractical.

### Thermal Perception Strategies

Another nutrition strategy that has received recent attention is the administration of orally-administered L-menthol, used to improve thermal perception rather than lowering body temperature per se [96]. This includes menthol mouth rinses [97], and menthol ingested with food [98]. A recent meta-analysis of menthol mouth rinsing (typically 25 mL of a 0.01–0.1% solution at regular intervals) found no significant physical performance benefits [97], in contrast to an earlier meta-analysis that found a small but beneficial effect on thermal sensation (Hedges’ *g* = -0.3, p = 0.004) and physical performance (Hedges’ *g* = 0.40, p = 0.003) [99]. Menthol in ingested foods and/or fluids has shown mixed results for physical performance, possibly due to differences in study methodologies. For example, a study of a menthol-enhanced energy gel (0.5% concentration) found no beneficial effects on time trial performance or thermal perception [98]; whereas a study of menthol beverage ingestion (25 mL of a 0.01% menthol solution every 5 min during EHS) found beneficial effects for running capacity compared to water [100]. Differences appear related more to the type of performance test utilized, with time trials (i.e., fixed distance, self-paced) less likely to show beneficial effects compared to time-to-exhaustion tests (i.e., fixed pace, variable distance/time) [96, 97, 99]. It is also important to recognize that any beneficial effects of menthol that result from improved thermal comfort may come at the risk of increased work output and core temperature, and that an altered relationship between thermal sensation and core temperature may place individuals at increased risk of EHI [11].

### Gastrointestinal Considerations

Considering the role of the gastrointestinal tract in the pathophysiology of heat stroke, there has been a recent, exponential growth in EIGS prevention and management research, especially in regard to exercising in hot ambient conditions. However, a large proportion of these studies contain substantial methodological limitations that may influence outcomes and interpretation, and subsequent translation into professional practice can be erroneous and/or unintentionally increase medical risk, as recently highlighted [19]. In this review, only studies that have met best practice standards for exercise gastroenterology research have been acknowledged and discussed. To date, EIGS prevention and management strategies have included targeted macronutrient (protein and/or carbohydrate) intake before and frequently during exercise, acute- or long-term dietary and/or nutritional supplement interventions, and EHS mitigating strategies (Table 1).

**Table 1:** Summary of proposed prevention and management strategies for EHS-associated disturbances to gastrointestinal integrity and/or subsequent gastrointestinal symptoms (GIS)

**Intervention**	**EIGS / Ex-GIS prevention and management impact**^**a**^
**Macronutrients** **(Pre and/or during EHS)**	- Carbohydrate, protein, and/or specific (i.e., L-citrulline) or mixed amino acid formulation intake before and frequently during exercise attenuates disturbances to gastrointestinal integrity and systemic responses
	- Carbohydrate intake before and frequently during EHS above individual tolerance levels disturbs gastrointestinal functional responses
	- Protein and carbohydrate intake before and frequently during exertional-heat stress above individual tolerance levels exacerbates GIS. Provision of a mixed amino acid formulation does not exacerbate GIS
	- No clear evidence for use of glutamine or arginine in mitigating exercise-associated disturbances to gastrointestinal integrity or GIS
**Dietary interventions** **(short- or long-term)**	- High FODMAP diet 24 h prior to EHS attenuates disturbances to gastrointestinal integrity, but with minimal impact on systemic responses. However, a high carbohydrate diet (~ 12 g/kg/day) for 48 h prior to EHS attenuates the magnitude of disturbances to gastrointestinal integrity and systemic responses
	- Low FODMAP diet results in lower burden on gastrointestinal functional responses compared with high FODMAP diet
	- Short term low carbohydrate, high fat diet exacerbates exercise-associated disturbances to gastrointestinal integrity and systemic responses, with possible GIS implications in response to exercise
	- No impact of gluten-free diet adapted by non-coeliac athletes, and acute low energy availability, on markers of EIGS in response to exercise
	- Low FODMAP diet results in lowered GIS severity compared with high FODMAP diet in response to EHS
**Nutritional supplement interventions** **(short- or long-term)**	Role in the prevention or management of exercise-associated perturbations to gastrointestinal integrity and/or GIS: i) Potential role^b^: - Prebiotics - Curcumin - Anthocyanins
	ii) No clear consistently beneficial role in the prevention or management of exercise-associated perturbations to gastrointestinal integrity and/or associated GIS: - Antioxidants - Bovine colostrum - Probiotics and syn-biotics - Nitrate
	iii) Potentially detrimental: - Antioxidant capsaicin - Probiotics
**Hydration**	- Starting exercise euhydrated mitigates exercise-associated disturbances to gastrointestinal integrity
	- Tolerable intakes of fluid during exercise to maintain euhydration mitigates exercise-associated disturbances to gastrointestinal integrity
	- Intolerance to high volumes of fluid intake to maintain euhydration exacerbates GIS
**Heat mitigating strategies**	**-** Heat acclimation protocols do not mitigate exercise-associated disturbances to gastrointestinal integrity and/or GIS. These outcomes are likely linked to modest acclimation protocols used within experimental procedures
	- Internal pre- and per-cooling mitigates exercise-associated disturbances to gastrointestinal integrity and/or GIS
	- External pre- and per-cooling mitigates exercise-associated systemic inflammatory responses. Unknown how external cooling strategies impact on gastrointestinal barrier integrity and GIS
	- Unknown how heat mitigating strategies impact gastrointestinal functional responses resulting from EHS

Contents within are extracted and summarized from Costa et al. (2017, 2020) [17, 18]

*EIGS* exercise-induced gastrointestinal syndrome, *Ex-GIS* exercise-associated gastrointestinal symptoms

^a^Marker outcomes in response to exertional or exertional-heat stress

^b^Provision of carbohydrate or protein before and frequently during exertional-heat stress out-performs beneficial effects purported with nutritional supplementation interventions

One of the most effective strategies to mitigate EHS perturbations to gastrointestinal integrity has been the intake of carbohydrate or protein (or derivatives, such as singular or amino acid mixtures) before and frequently during exercise. Such strategies have attenuated intestinal epithelial injury and permeability, luminal pathogenic translocation, and systemic inflammatory responses, without exacerbating GIS when consumed within individual tolerance levels [18, 25, 101, 102]. Moreover, a recent study reported EIGS attenuation with two amino acid formulations consumed for 7-days before and frequently during 2 h EHS, showing superior protection over previous singular amino acid supplementation (e.g., glutamine, L-citrulline, and arginine) [101]. Nevertheless, tolerance to macronutrient feeding during EHS is a prime factor for the onset and severity of GIS, considering the situation of compromised gastrointestinal functional responses [22, 35, 103, 104]. Therefore, factors such as intake volume, type, frequency, nutrient quantity and quality, and individual characteristics (i.e., gastric emptying, orocecal transit time, nutrient digestion and absorption), need to be considered when prescribing macronutrient intake during exertional-heat stress for the management of EIGS.

From a whole-diet perspective, although gluten-free diets appear to have no impact on EIGS outcomes [105], acute dietary fermentable oligo- di- mono-saccharide and polyol (FODMAP) manipulation has been reported to provide some beneficial effects. Although a 24 h low (2 g FODMAP/day, 6.0 g CHO/kg BM/day) FODMAP diet supported a reduction in EHS-induced GIS, the 24 h high (47 g FODMAP/day, 5.2 g CHO/kg BM/day) FODMAP diet appeared protective against EIGS, by attenuating intestinal epithelial injury and a more favorable systemic bacterial endotoxin profile [36]. However, a more recent study showed that increasing total dietary carbohydrate (9.5 g CHO/kg BM/day) for 48 h before exertional stress, irrespective of the FODMAP content (2 vs 51 g FODMAP/day), ameliorates exercise-associated perturbations to gastrointestinal integrity, supporting the strategy of carbohydrate provision per se in the management of EIGS to EHS [106]. Furthermore, a 6-day ketogenic, low carbohydrate high fat (LCHF) diet has recently been shown to exacerbate intestinal epithelial cell injury, bacterial translocation, and system inflammatory responses prior to and after a 25 km simulated race walk, compared with high carbohydrate and low energy availability diet [107, 108]. These outcomes are likely due to the intestinal epithelial hyperpermeability associated with fat digestion and absorption, which inevitably promotes pathogen translocation into circulation, inducing local and systemic inflammatory responses [109]. These results pose a concern considering the recent popularity of LCHF diets amongst sporting (e.g., ultra-endurance athletes) and occupational (e.g., military) populations, that adherence to such diets prior to EHS may increase risk for clinical consequences of EIGS.

There has been considerable investigation into proposed acute or long-term, non-macronutrient nutritional supplement interventions for prevention or management of EIGS, including, but not limited to: antioxidant and/or anti-inflammatory agents, biotics (i.e., pre-, pro-, syn-biotics), bovine colostrum, curcumin, and nitrate [17–19, 110]. Due to the limited number of studies and/or small sample sizes for each supplement type, contradictory conclusions between studies, substantial methodological issues previously reported, no or modest outcomes in only one or a few EIGS pathophysiological biomarkers, and some negative outcomes reported, to date, no acute or long-term nutritional supplement intervention has provided consistent, clinically relevant, beneficial outcomes that warrant recommendation or application in practice for the prevention and management of EIGS in response to EHS.

Considering EIGS pathophysiology has been associated with (and can be predicted by) increased core temperature, with maximal core temperature ≥ 39.5 ºC aligned to EIGS biomarkers of clinical relevance [9, 26], it is not surprising that various cooling strategies (i.e., internal or external pre- and/or per-cooling strategies) have been investigated in an attempt to prevent or manage EIGS and associated GIS [12, 18]. As previously discussed, hypohydration exacerbates EIGS and associated GIS, linked to greater thermophysiological strain; albeit explored in temperate conditions [27]. Secondly, while not a nutrition strategy per se, heat acclimation may provide some gastrointestinal integrity protection against EHS, but existing studies lack sufficient exposure and clear acclimation outcomes to confirm benefit [12, 111–113]. Although in its infancy, both internal and external pre- and per-cooling strategies have been investigated with regard to EIGS, reporting some degree of attenuation of intestinal epithelial integrity disturbance, systemic inflammatory responses, and/or GIS [95, 114]. Studies reporting no beneficial effect appear to lack sufficient EHS and EIGS perturbation, and/or provided beverages containing carbohydrate [115]. Such methodologies fail to meet the minimal detectable change for EIGS biomarkers and provide artefact protection via carbohydrate provision [19, 26].

In accordance with published evidence using ‘best practice’ methodologies [19], recommendations to prevent or manage EIGS, and associated GIS, in response to EHS include: consuming tolerable carbohydrate, with or without protein (or amino acid mixture formulations) before and frequently during EHS; consume a high carbohydrate, low FODMAP diet the day before exposure; adhere to euhydration drinking practices before and during exposure; and utilize strategies that attenuate the rise in core body temperature during EHS.

### Renal Considerations

The established mechanisms for AKI in response to EHS mostly overlap with those that influence the risk of impaired performance, EHI, and EIGS. In particular, the fluid, sodium and cooling strategies already discussed are likely to reduce the risk of AKI also [44]. An additional nutritional consideration, not specific to EHS per se but possibly relevant regarding fluid replacement, is a recent hypothesis that sugar-sweetened beverages, and particularly the intake of fructose may contribute to decrements in renal function in a dehydrated state, due to high quantities of fructose being partly metabolized in the kidneys, with subsequent uric acid production and localized oxidative stress [116]. Until recently this hypothesis was supported only by evidence from epidemiological studies of humans [117], and intervention studies in rodents [116]. One recent study however, investigated soft-drink intake in humans on biomarkers of renal function and damage, concluding that compared to plain water, soft drink consumption during and post-exercise increased these biomarkers (serum creatinine and neutrophil gelatinase associated-lipocalin, NGAL) [118]. These findings require care with interpretation due to the lack of an isocaloric control condition. A comparison to water only cannot demonstrate the independent effect of fructose or other components of the test beverage (relative to other carbohydrate types or macronutrients) on renal function, nor can it exclude the possibility of a higher serum creatinine concentration resulting from a greater work output in the soft drink trial, due to greater carbohydrate availability during the maximal effort occupational activity simulations [118]. Further research is needed to demonstrate whether the observed effect is due to the beverage formulation, the presence of any macronutrition, or differences in physical work output, especially since the use of carbohydrate-containing beverages generally, and those containing fructose specifically, have been expressly recommended during exercise to optimize physical performance [119, 120], and post-exercise to rapidly restore muscle and liver glycogen content and improve subsequent performance [121–123].

## Conclusion

The impact of climate change on both athletic and occupational health and performance necessitates practical application of strategies to minimize the risk of EHI, EIGS, AKI, and optimize physical performance outcomes in EHS conditions. Optimizing hydration and sodium status before and during EHS, in addition to internal cooling with ice, all have promising practical application. However, each approach also comes with practical limitations that must be overcome to realize their efficacy. A high carbohydrate, low FODMAP diet the day before EHS exposure, and frequent provision of carbohydrate and/or protein during EHS, may also help to prevent and manage EIGS, which poses greatly increased risk in hot ambient conditions. Most published studies of these nutritional strategies are focused on athletic rather than occupational settings, and this should be a focus for future research given the predicted increased in occupational heat exposure, and potential for lost productivity and heat related illness on a warming planet.

**Fig. 1:**
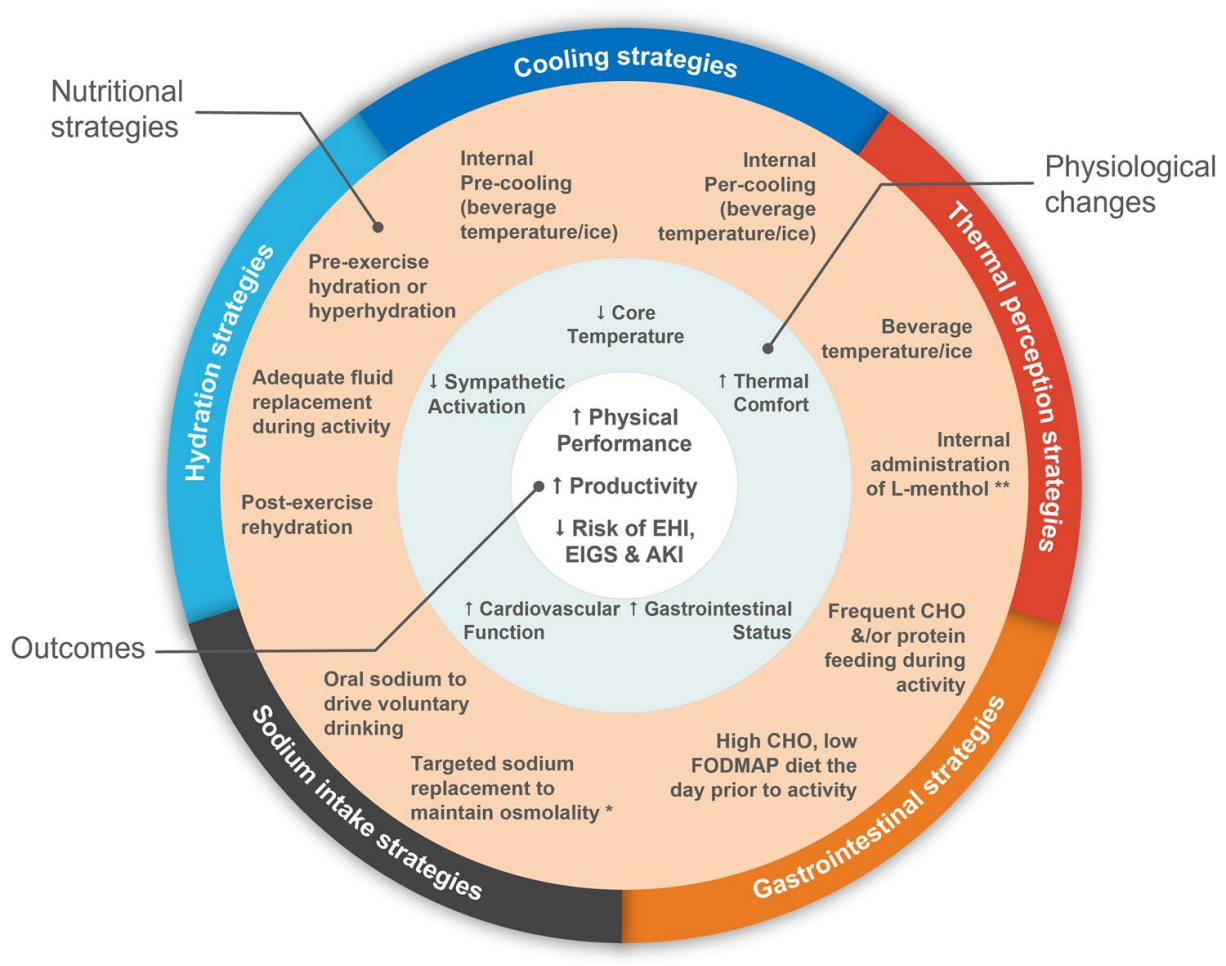
Summary of nutritional strategies that aim to improve physical performance and productivity, and/or reduce the risk of heat-related health consequences during exertional-heat stress. AKI: Acute kidney injury, CHO: Carbohydrate, EHI: Exertional heat illness, EIGS: Exercise-induced gastrointestinal syndrome. * Targeted sodium replacement is only necessary to maintain osmolality when fluid replacement is significant (> ~ 70% of losses). ** Use of L-menthol can improve thermal perception, but in the absence of a true change in core temperature, may increase the risk of EHI

## Data Availability

No datasets were generated or analysed during the current study.
